# Preface: mechanical perturbations induced quench: a challenge of superconductor mechanics

**DOI:** 10.1093/nsr/nwad044

**Published:** 2023-03-03

**Affiliations:** Research Center for Applied Mechanics, Xidian University, China

Superconducting materials have been gradually applied in many areas including energy, transportation, medical imaging and communication. Among them, superconducting magnets that can generate a strong magnetic field above 10 Tesla with absolute advantages of higher quality, lower energy loss, and smaller volume, have become key to the performance improvement of cutting-edge science and large-scale engineering devices such as high-energy accelerators, thermonuclear fusion reactors, and magnetic resonance imaging devices [1]. However, in the development of hybrid test magnets with the highest field of 45.5 T [2] and all-superconducting magnets with the highest field of 32.35 T [3] in the world, it has been found that, although the ambient temperature, magnetic field and current intensity of the superconducting material do not exceed the critical values of its superconducting state, the magnet still shows significant performance degradation and even quench. It is worth mentioning that, although scholars in the field of electrical engineering also carried out mechanical analysis during the design and development of high-field superconducting magnets, they checked the strength of materials and structures, and explored stress management techniques to mitigate conductor degradation. But they did not realize that these mechanical responses are also a major cause of quench in superconductors. This kind of quench caused by the mechanical response, now referred to as ‘mechanical perturbations induced quench’, was first proposed by Prof. You-He Zhou, an academician of the Chinese Academy of Sciences and president of the Institute of Superconductors Mechanics, Lanzhou University. Prof. Zhou and his research team have carried out over 20 years of research in the field of superconductor mechanics (SM). Their work on basic experiments of SM, development of testing instruments for mechanical behavior of superconducting materials, theoretical modeling and quantitative analysis are unique and leading.

This special topic includes a review, an interview and two research papers, whose main purpose is to introduce the concept and connotation of ‘mechanical perturbations induced quench’, and the latest research progress, including studies of alleviating the brittleness of superconducting materials, and the influences of cracks on the thermomagnetic instability, which is often considered a reason for quench.

The authors of the review article are Prof. You-He Zhou and Prof. Y. Iwasa, who are from Lanzhou University and Massachusetts Institute of Technology, respectively. They reviewed the research progress and challenges of fundamental mechanical problems in high-field superconducting magnets [4]. In this issue, it is a great honor to interview Prof. Jian-gang Li, an academician of the Chinese Academy of Engineering. Prof. Li emphasized that, during the development of fusion energy–oriented high-field superconducting magnets [5], the influence of mechanics on superconductivity is the fundamental issue and difficulty.

The first research paper in this special issue comes from Lanzhou University. Zhang *et al*. [6] have constructed the layered interlocking microstructure to greatly improve the toughness of YBa_2_Cu_3_O_7-x_ (YBCO) superconductors, without reducing its superconducting properties, based on the customized 3D printing combined with a freeze-drying process. The second research paper in this special issue comes from Prof. Ze Jing of Northwestern Polytechnical University. Prof. Jing has been selected into the Young Elite Scientists Sponsorship Program of the China Association for Science and Technology, and won the first prize of the Jan Evetts SUST Award by the international journal *Superconductor Science and Technology* in 2020. Prof. Jing focuses on the effects of edge cracks on the thermomagnetic instability (TMI) of Type-II superconducting films and finds that the threshold field, the avalanche patterns, and the multifractal spectrum of the TMI process transit with the extension of the edge crack [7].

It can be seen from the above articles and interview that, on the one hand, SM is extremely important for the development of high-field superconducting magnets; on the other hand, the influence of mechanics to superconductivity, is not only part of the traditional understanding of superconductivity degradation, but also central to the new concept of ‘mechanical perturbations induced quench’. It is hoped that through the publication of this special issue of SM, electrical engineers can better understand the nature of ‘mechanical perturbations induced quench’, and continue making progress in developing high-field superconducting magnets.

## Notes

Editorial Board Member of NSR
